# Diabetes (*db/db*) mutation-induced endometrial epithelial lipoapoptosis: Ultrastructural and cytochemical analysis of reproductive tract atrophy

**DOI:** 10.1186/1477-7827-3-15

**Published:** 2005-04-27

**Authors:** David R Garris

**Affiliations:** 1 Division of Cell Biology and Biophysics, School of Biological Sciences, University of Missouri-Kansas City, Kansas City, Missouri 64110 USA

## Abstract

**Background:**

The diabetes (db/db) mutation in C57BL/KsJ mice promotes a progressive cytolipidemia within the endometrial epithelial (EE) layer of the female reproductive tract which results in premature cellular and organ atrophy. The current studies focus on the ultrastructural and cytochemical changes which promote nuclear apoptosis and cytostructural disruption following the expression of endometrial hypercytolipidemia which promotes diabetes-associated organoinvolution and manifest infertility.

**Methods:**

Control (normal:+/+) and diabetes (db/db) genotype groups were prepared for high resolution light microscopic analysis of cytolipidemia and nuclear apoptosis (TUNEL-labeled 3'-DNA fragmentation) indices and compared to the transmission electron (TEM) microscopic analysis of endometrial tissue samples collected from 8–16 week-old groups.

**Results:**

Compared to controls, db/db mutation expression induced a dramatic increase in EE cytolipid vacuole volume and density within the epithelial endometrial layer. TEM analysis revealed that cytolipid vacuole accumulations initially aggregated at the baso-polar regions of UEE cells in response to the systemic hyperglycemic/hypertriglyceridemic conditions which characterized the (db/db) groups. Progressive cytoplasmic movement of the lipid pools into perinuclear compartments of affected EE cells induced nuclear isolation from organelles that were displaced towards peripheral cytoplasmic compartments. Cytochemical analysis of lipid vacuole accumulations indicated attraction towards, and incorporation within, the nuclear envelope of hyperlipidemic cells. Co-localization of nuclear apoptotic 3'-DNA fragments within identified hyperlipidemic EE cells was coincident with the cytochemical and ultrastructural identification of lipid penetration through the nuclear envelope in db/db mutants.

**Conclusion:**

These results are the first cytochemical indication that the metabolic disturbances in db/db mutants which promote hypercytolipidemia are coincident with lipoapoptosis-induced nuclear dissolution, as denoted by DNA fragmentation analysis. The lipidemia-induced alterations in intracellular organelle and nuclear architectures suggests that the metabolic disturbances in glucose and lipid metabolic cascades in diabetes (db/db) mutants disrupts cytointegrity, culminating in nuclear disregulation (as indicated by lipoapoptosis) and eventual premature reproductive tract organoinvolution and resultant, manifest, reproductive sterility.

## Background

The cytoarchitecture of the female reproductive tract is severely compromised by the deleterious influences of diabetes-induced alterations in utero-ovarian cellular glucometabolism [1-3]. In humans [4-6] and experimental models [7-11], diabetes-associated alterations in uterine endometrial metabolism and structure have been associated with pronounced hypercytolipidemia, a hyper-caloric metabolic response that induces cellular hyperlipidemia and subsequent promotion of premature reproductive tract involution [2,3,12-16]. The resulting reproductive incompetence is characterized by reproductive acyclicity [13,14], compromised ovarian follicular development [14], depressed ovarian steroid hormone synthesis [17], depressed sensitivity and responsivity to endocrine stimulated cellular metabolism [18-20] and enhanced utero-epithelial atrophy [2]. The affected endometrial architecture is characterized by an enormous increase in intra-and inter-cellular lipid depositions [2,13], resulting from the interstitial perivascular escape and imbibition of elevated systemic triglyceride and free fatty acid moities [21,22] which characterize the overt diabetes (Type 2) metabolic (X) syndrome [23,24]. Ultimately, exposure to the chronic influences of the non-homeostatic metabolic condition induces a lipoatrophy syndrome [12-14], characterized by the progressive accumulation of cytolipid inclusions [2], organelle dissolution [2], nuclear compartment isolation [15], suppressed cellular oxidative metabolism [13], and cyto-atrophy [9,13,14,17]. Recent reports have indicated that the expression of the diabetes (db/db) mutation in C57BL/KsJ mice compromises reproductive tract maturation by promoting hypercytolipidemia within the endometrial epithelial (EE) layer [2] that is characterized by a progressive lipid-isolation of the cell nuclei from surrounding cytoplasmic organelle compartments [15]. The expanding endometrial cytolipid volume in db/db mutants has been associated with disrupted nuclear chromatin (DNA) structural integrity and pycnosis-associated degeneration [24]. However, the co-incident expression of metabolic hypercytolipidemia and structural nuclear dissolution, as indexed by 3'-DNA fragmentation [24] within apoptotic nuclei, remains to be demonstrated. The present studies were designed to evaluate the co-incident cytochemical and ultrastructural alterations which promote premature, progressive lipoapoptotic nuclear degeneration within the endometrial epithelial tissue layer of the obese, hyperglycemic, hyperlipidemic and hypogonadal (infertile) db/db-mutant reproductive tract.

## Materials and methods

### Animals

Adult, female C57BL/KsJ mice (Jackson Laboratory, Bar Harbor, ME), between 8 and 16 weeks of age, denoting the overt and chronic phases of the Type 2 diabetes syndrome [14], were used in these studies and maintained in accordance with the National Institutes of Health guidelines for the care and use of laboratory animals (NIH publication no. 80-23). Littermate controls (+/+) and diabetes (db/db)-mutant genotypes, were pair matched for phenotype, tissue sampling and blood glucose concentration comparisons during the course of these studies. All mice were housed five per cage, grouped according to genotype, under controlled environmental conditions (23°C), with an established photoperiod of 12 hr light/day (lights on: 0600 h) [13,14]. Blood glucose levels (Ames Glucometer method), serum triglyceride concentrations (Sigma, St. Louis) and body weights were monitored for each of the 8 to 16-week-old age groups as previously described [13,14]. Animals exhibiting both obesity (≥25 grams) and pronounced hyperglycemia (≥200 mg/dl) and hyperlipidemia (≥ 200 mg/ml serum triglycerides) relative to controls (≤150 mg/dl and mg/ml, respectively) were considered as overt, obese-diabetics [16,23], with the comparative expression of these indices noted relative to control or genotypic mutation (Table I) groups throughout the experimental period.

**Table 1:** Phenotype, Uterine Tissue Biomass and Glycemia Indicators of Diabetes (db/db) Mutation-Induced Alterations in C57BL/KsJ Mice.

**Index**	**N**	**Groups**		**P < :**
			
		**Control (+/+)**	**Diabetes (db/db)**	
Body Weight (g)	5	21 ± 3	47 ± 5	0.001
Blood Glucose (mg/dl)	5	103 ± 8	428 ± 16	0.001
Uterine Weight (mg)	5	44 ± 3	13 ± 4	0.01
Serum Triglycerides (mg/ml)	5	143 ± 8	312 ± 24	0.001

All values for the indicated parameters are represented as group (N) means (± SEM) for control (+/+) and diabetes (db/db)-mutant C57BL/KsJ mice, with statistical intergroup differences (P ≤) indicated.

### Tissue Collection and Preparation

Uterine endometrial tissue samples from each group of control (+/+) and diabetic (db/db) matched-paired genotypes were collected, weighed and prepared for high resolution light microscopy (HRLM), cytochemical analysis of cytoplasmic lipid depositions and transmission electron microscopic (TEM) examination as previously described [2]. In brief, mice were anesthetized at 8 (i.e. overt phase) or 16 weeks (i.e., the chronic phase of Type 2 syndrome expression and reproductive tract compromise) [14] with sodium pentobarbital and systemically perfused with 50 ml of physiological saline and 100 ml of Karnovsky's fixative solution. Collected mid-cornua uterine tissue samples were cleaned, blotted, blocked and embedded in either paraffin or plastic using conventional techniques [2]. All tissue samples were subsequently sectioned and stained with a toluidine blue-basic fuchsin mixture for polychromatic identification [24,25] of cellular lipid pools by HRLM or with osmium tetroxide [2] prior to examination by TEM.

### High Resolution, Digital and TEM, Hypercytolipidemia Analysis

Tissue sections prepared for light microscopic analysis were used for polychromatic organelle differentiation, the localization of intracellular lipid inclusion accumulations, and the determination of cytoplasmic changes associated with the progressive expression of the db/db mutation as previously described [25]. Photographic images of uterine tissue compartments and epithelial cell populations from the prepared tissue samples were captured with an Olympus (Olympus Optical, Tokyo, Japan) digital graphics camera and microscope unit, with lipid vacuole pools digitally enhanced utilizing polychromatic stain identification, and digital-color scale conversion for chemical-specific triglyceride localization analysis [25]. Tissue sections prepared for TEM analysis from the same groups were analyzed for structural variations in cytoplasmic changes in organelle and lipid inclusion density, as well as for uterine basal lamina and peri-nuclear changes induced by the expressed hypercytolipidemia associated with the expression (db/db) mutation [2].

### Localization and Analysis of db/db-Associated Nuclear Lipoapoptosis

Uterine samples from the designated groups were collected and rapidly frozen for cryostat (-20°C) sectioning then subsequently prepared for TUNEL (FD NeuroTechnologies; Ellicott City, MD) labeled apoptotic 3'-DNA fragmentation analysis, a recognized chemical marker associated with nuclear chromatin dissolution, as previously described [20]. Slides were placed in 0.1 M phosphate buffer (PBS: pH 7.4) containing 4% (v/v) paraformaldehyde for 30 minutes. Tissue sections were subsequently washed (x2 rinses @ 5 minutes each) in 0.01M PBS, then fixed in pre-cooled (-20°C) ethanol:acetic acid (2:1 v/v) for 5 minutes, washed (x2) in PBS for 10 minutes to assure proper tissue preservation prior to preparation for TUNEL labeling. Detection of free 3'-hydroxyl terminus DNA fragments was performed using the *in situ *TdT-mediated dUTP-biotin nick end labeling (TUNEL) technique. Terminal deoxynucleotidyl transferase was utilized for the catalyzed incorporation of biotinylated deoxyuridines onto the exposed 3'-hydroxyl termini of DNA fragments which labeled apoptotic cells. The integrated biotins were enhanced and visualized as dense, localized avidin-biotin-complexes identifiable by HRLM. All TUNEL labeled endometrial samples were subsequently counterstained for polychromatic cytostructural analysis [25], allowing for the identification of hypercytolipidemia within the same cells labeled for nuclear apoptosis. Intracellular nuclear or cytoplasmic organelle (mitochondrial) TUNEL-label specificity was evaluated prior to cytochemical analysis of co-localized perinuclear lipid vacuole density profiles and 3'-DNA fragments, indicative of apoptotic cytodegenerative alterations, in affected endometrial epithelial cells exhibiting hypercytolipidemia.

### Statistical Analysis

Values for body weights and blood glucose concentrations were expressed as group means (± SEM) for the designated genotype groups. Intergroup differences were determined using the Student's T-test and Analysis of Variance exams, with a p ≤ 0.05 accepted as representing statistical intergroup measurement differences.

## Results

### Cytochemical and Ultrastructural Analysis of Endometrial Epithelial Hypercytolipidemia

The changes in body weights, uterine weights and blood glucose concentrations in C57BL/KsJ mice resulting from the expression of the diabetes (db/db) mutation are indicated in Table I. Dramatic increases in phenotypic obesity and associated hyperglycemic conditions characterized (db/db) groups relative to (+/+) indices. In contrast, uterine weights in the db/db-mutant group decreased (Table I) in association with the progressive endometrial hypercytolipidemia and atrophy which characterized the uterine samples prepared for HRLM and TEM analysis.

**Figure 1:**
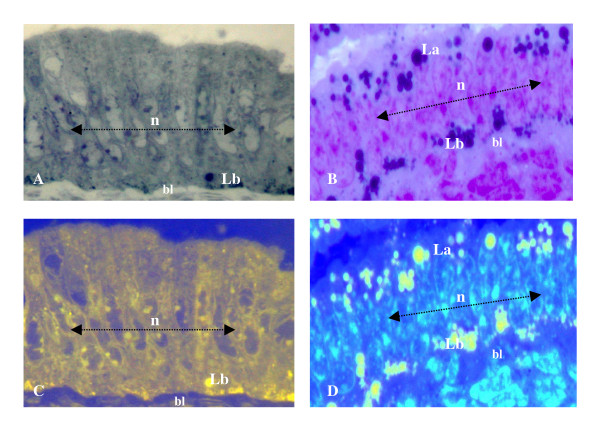
Photomicrographic (x400) comparisons of representative control (+/+:A) and diabetes (db/db)-mutant (B) uterine endometrial epithelial tissue layers by HRLM analysis, depicting the normal, sub-nuclear (n: arrow line) basal pole lipid (Lb) pools typical of +/+ groups located adjacent to the underlying stromal basal lamina (bl), as compared with the dramatic expansions of Lb and apical pole lipid (La) pools in db/db (B) groups. By digital enhancement of cytochemical lipid (triglyceride) pools in +/+ (C) and db/db (D) groups, the sub- and supra-nuclear cytoplasmic lipid pool expansions in db/db groups (D) were accentuated relative to the cytoplasmic volume and distribution patterns of lipid vacuoles in control specimens (C). (Technical Note: Enzymatic incubation of tissue sections, as described in the Materials & Methods section, for cytochemical and TUNEL-labeling is responsible for a moderate reduction in image resolution presented in Figures 1, 4, and 5, represents an intrinsic technical limitation associated with co-localization analysis of hyperlipidemia and apoptosis indices within tissue preparations.)

**Figure 2:**
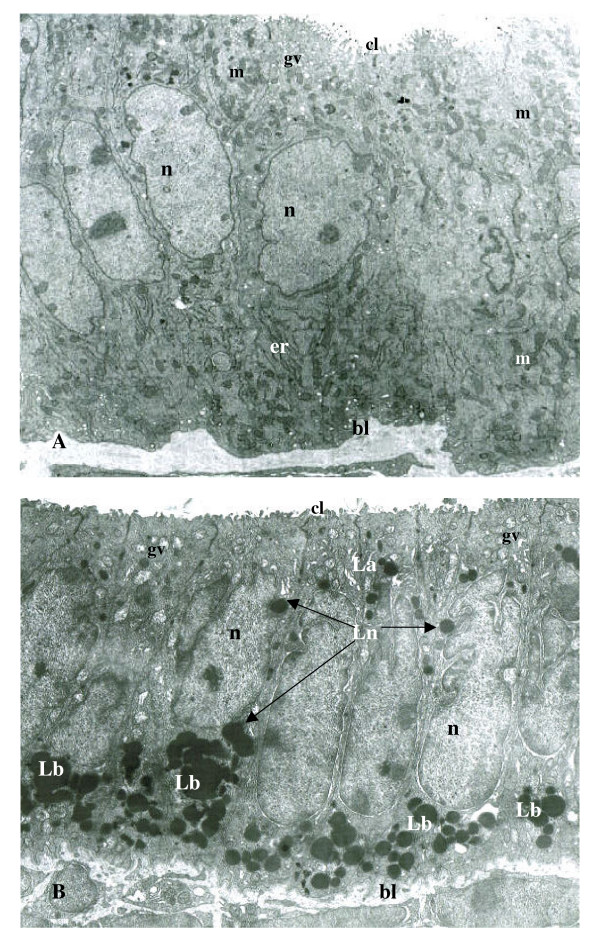
Ultrastructural (× 7350) analysis of control (A: +/+) endometrial epithelial tissue cells indicated prominent central nuclei (n) supported by a well-defined smooth stromal basal lamina (bl) and a cytoplasm possessing a rich organelle population including abundant mitochondrial (m) and prominent rough endoplasmic reticulum (er) compartments in the basal pole regions, as well as a well-developed Golgi vesicular apparatus (gv) and surface ciliary (cl) arrangement in the apical zones. In contrast, the endometrial epithelial cell layers of diabetes (db/db) mutant groups (B) were characterized by a convoluted bl membrane and a sub-nuclear (n) region dominated by enormous concentrations of basal pole lipid (Lb) vacuoles. Intracytoplasmic migration of the lipid into the perinuclear compartment (Ln), as well as a prominent reduction in basal cytoplasmic organelle populations, occurred in conjunction with nuclear envelope convolutions, with internal nuclear membrane invaginations associated with Ln depositions (arrows). Apical pole lipid (La) vacuole densities were prominent in db/db epithelial tissue samples, and were associated with expanded Golgi vesicular (gv) cisterns and blunted apical ciliary (cl) arrays as compared with +/+ structural (A) indices.

**Figure 3:**
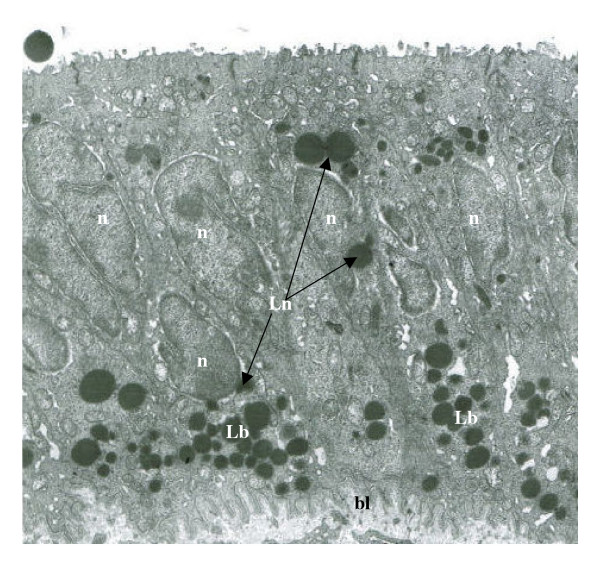
Transmission electron microscopic (× 6600) analysis of the progressive alterations induced within the endometrial epithelial cell layers of db/db mutants relative to expanding intracytoplasmic lipid pools in the basal (Lb) and perinuclear (Ln) compartments of affected cells. The prominent convolutions of the basal lamina (bl) of cells demonstrating enormous Lb accumulations were coincident with the recognized expansion of the perinuclear space (pns) in regions associated with Ln contact, or approximation with, the external nuclear envelope (arrows). The prominent nuclear membrane contact by Ln vacuoles occurred along all nuclear membrane planes, often in association with membrane invagination into the nuclear (n) compartment.

**Figure 4:**
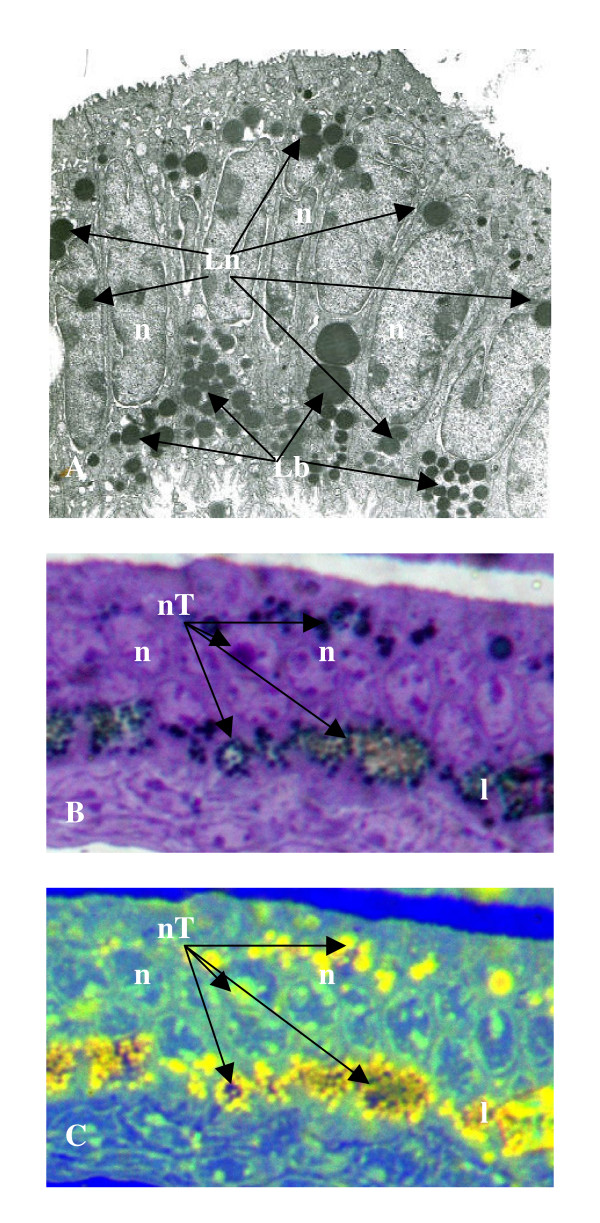
Ultrastructural (A: ×3750) and cytochemical (B-C: ×400) analysis of endometrial hypercytolipidemia depositions within epithelial cells of db/db mice localized within the basal (Lb) and perinuclear (Ln) compartments of affected cells. Digital enhancement (B) of cytoplasmic lipid vacuole accumulations (l) below the nuclear (n) layer, and within the perinuclear space (arrows), indicated intense TUNEL-indicated (black stain moieties) fragmentation of 3'-DNA components localized within the mitochondria-rich basal pole region as well as the nuclei (nT) of cells exhibiting lipoapoptosis. The cytochemical co-localization (C) of cytoplasmic lipid pools (yellow fluorescence) with TUNEL-labeled nuclear apoptotic DNA fragments (nT) within the same endometrial epithelial cell populations of db/db mutants indicated the coincident relationship between metabolic hypercytolipidemia and nuclear apoptosis-induced cytodissolution which resulted in uterine involution (Table I) and reproductive sterility.

The accumulation and retention of EE cell cytolipid stores by (+/+) tissues was found to be restricted to the baso-polar regions of all control samples examined by HRLM (Figure 1A, C) and TEM (Figure 2A) analysis between 8 and 16 weeks of age. The chemical-specific localization of basal cytoplasmic triglyceride deposits characterized +/+ endometrial epithelial cells, in which cytoplasmic organelle distribution and organization were indicative of a viable cytoarchitecture (Figure 2A). In contrast, the polychromatic identification of hypercytolipidemic depositions in (db/db) tissue samples was demonstrated by the cytoarchitectural alterations noted by both HRLM cytochemical (Figure 1B, D) and TEM (Figure 2B) analysis. Characteristic of all 8 – 16 week old db/db-mutant tissue samples (Figures 2B; 3) was the enormous increase in cytoplasmic triglyceride pools which were distributed in a prominent gradient pattern between basal, perinuclear and apical cytoplasmic regions (Figures 2B,3). TEM analysis of endometrial epithelial samples from db/db groups indicated that the basal pole cytoplasmic compartments contained dense, expanded lipid vacuole accumulations which progressively migrated to surround and occupy the perinuclear space of affected cells (Figure 3). The progressive changes in nuclear envelope configurations (i.e., convoluted membrane, pycnotic distortions) induced by the perinuclear lipid pool expansions included dramatic invaginations of the external nuclear membrane, chromatin clumping along the inner nuclear membrane and physical disruption of the envelope integrity in regions where lipid moieties were observed to contact and penetrate the outer nuclear lamina (Figures 2B, 3, 4A) as demonstrated by TEM analysis. The nuclear membrane and chromatin disturbances associated with the pronounced perinuclear lipid accumulations were further characterized by a prominent expansion of the perinuclear space surrounding each affected cell (Figure 3), promoting a physical isolation of the nuclear compartment from the cytoplasmic organelles which were peripherally displaced by the expanding cytoplasmic lipid pools.

### Cytochemical and TUNEL-Label Analysis of Nuclear Lipoapoptosis

**Figure 5:**
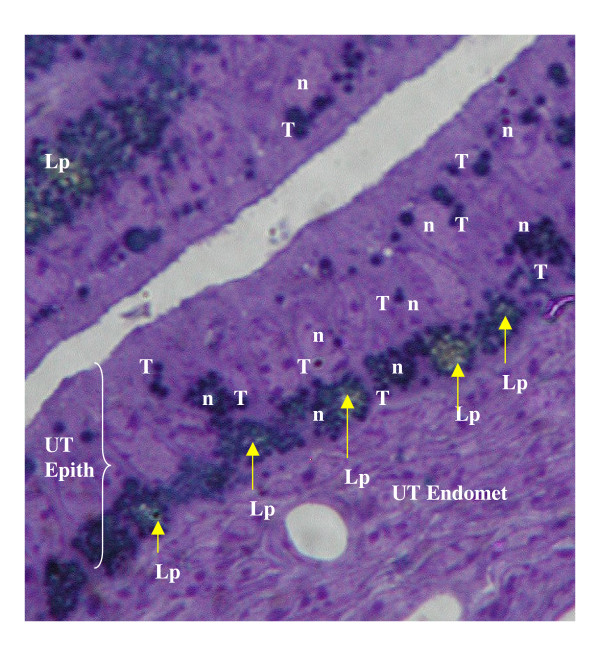
Photomicrograph (× 2000) of the db/db uterine endometrial epithelial (EE Layer) cell layer and underlying endometrial stroma (UT Endomet) demonstrating the coincident events of hypercytolipidemia (Lp: yellow fluorescent depositions) and TUNEL-labeled (T: black stain indicator) nuclear (n) co-localized within affected cells. The infiltration of the nuclear envelope by hyperlipidemic vacuole pools was denoted in epithelial cells that demonstrated ultrastructural (Figures 3-4) or cytochemical (Figure 4) indicators of apoptotic cytoatrophy.

The influence of db/db-induced hypercytolipidemia on nuclear disruption was evaluated by the combined cytochemical localization of intracellular triglyceride depositions by computer-assisted, digital cytochemical analysis and the co-localization of 3'-DNA fragmentation by TUNEL-labeled counterstaining as an index of nuclear chromatin dissolution (apoptosis) events (Figure 4B–C). TUNEL-indexed nuclear apoptosis was localized in db/db UEE cells with co-incident hypercytolipidemic vacuolar expansion (Figure 4B). When subjected to triglyceride cytochemical analysis (Figures 4C, 5), nuclear TUNEL-label was co-localized within the cytolipid depositions present in both the mitochondrial-rich basal pole cytoplasmic compartment, as well as within the perinuclear and nuclear compartments of affected db/db cells (Figure 4C). Dense nuclear TUNEL-label was located within both the defined nuclear compartments of db/db cells (Figure 4C), and was prominent within the basal cell layer of proliferating epithelial tissue in which both TUNEL and hypercytolipidemic vacuole depositions were co-localized (Figure 5). In db/db EE samples demonstrating perinuclear lipid pool penetration of the affected nuclear envelope, TUNEL-indicated lipoapoptosis was a consistent index of cellular compromise promoted by triglyceride penetration and disruption of epithelial nuclear organization (Figure 5). The lipometabolic disruption of cytostructural organization was coincident with the co-localization of nuclear apoptosis (TUNEL-label) and hyperlipidemic, cytochemical indices, which characterized the premature EE cytoatrophic involution of the female reproductive tract associated with the overt expression of the type 2 (NIDDM) diabetes syndrome.

## Discussion & Conclusions

**Figure 6:**
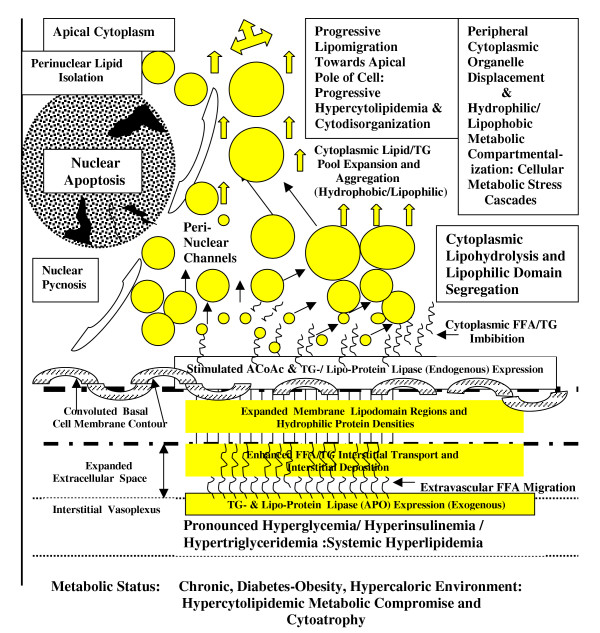
Diagrammatic representation of the cumulative influences of *chronic *diabetes-obesity syndrome influences on the progressive cytotransformation into hyperlipidemic cell types, eventually results in lipoidal infiltration into, and dissolution of, the nuclear chromatin (DNA) matrix continuity culminating in lipoapoptosis and premature cytoatrophy.

The current results demonstrate that the hyperlipidemic metabolic microenvironment induced by the expression of the diabetes (db/db)-mutation promotes a progressive lipoapoptotic cytoatrophy of EE tissue, events which contribute to premature organoinvolution of the female reproductive tract and manifest sterility in the C57BL/KsJ murine model of a gene-mutation linked, inherited, dysregulated metabolic syndrome [12,23]. The unique co-localization of dense cytolipid vacuole pools in affected cells experiencing TUNEL-indexed nuclear apoptosis and chromatin dissolution indicates that the hypercytoplasmic sequestration of extracellular lipids into the cytoplasmic perinuclear compartment compromises nuclear organization by the promotion of DNA fragmentation and subsequent nuclear degradation by lipo-infiltration (Figure 6). Ultimately, the progressive cytometabolic disruption of the endometrial layers compromises reproductive tract cytostructural and tissue integrity [2,3], as indicated by the reduced uterine biomass in db/db groups. Similar to diabetes- and obesity-associated reproductive complications in human clinical studies [4,6], the recognized alterations in both phenotypic and cytolipid (metabolic) indices induced an adipose-like cellular organization within the EE layer [2]. The resulting changes in intracellular organelle displacement towards peripheral cytoplasmic compartments, the blunting of apical EE ciliary and microvillus expressions [2], the altered chemical (i.e., hyperlipidemia) composition and the coincident nuclear apoptotic dissolution (Figure 6), correlated with the recognized functional compromise and premature organo-involution of the female reproductive tract [2] in db/db genotype mutants. The progressive intracytoplasmic trafficking of intracellular lipid pools from basal-to-perinuclear-to-apical cytoplasmic loci, has been recognized to be associated with both the duration and severity of the systemic metabolic aberrations in db/db-mutants [2,15]. These collective data suggest that the progressive lipid infiltration of the EE layer promotes the indicated structural, metabolic and lipoapoptotic disruption of nucleus (DNA)-directed transcriptional metabolic cascades that ultimately induce non-homeostatic cytoarchitectural changes in affected db/db cells which become incapable of supporting normal reproductive tract function (Figure 6). The progressive disruption of these structural indices and interdependent metabolic cascades culminates in the resultant, cumulative, cytoatrophic premature organoinvolution of the female reproductive tract and manifest sterility.

Of particular interest was the progressive perinuclear accumulation and infiltration of the nuclear compartment by db/db-mutation associated expansion of cytoplasmic lipid pools. By both HRLM and TEM analysis, the identification of lipid vacuole contact with, or infiltration through, the nuclear envelope was evidenced in cells exhibiting TUNEL-labeled lipoapoptosis. Progressively, the lipid migrations accumulated as low density lipid vacuole pools in the perinuclear space, but progressively migrated into contact with the external nuclear envelope and expanded extranuclear cytoplasmic space. Subsequent isolation of centric nuclei by lipid infiltration into the perinuclear space (Figures 3,4) was accompanied by the induction of prominent nuclear envelope pycnotic convolutions that were associated with lipid vacuole migration into contact with, or through, the external nuclear lamina. The translaminal migration and intranuclear lipid depositions (Figure 6) occurred in association with coincident TUNEL-indexed DNA fragmentation. These observations suggest that the hypercytolipidemic metabolic condition promotes a lipid-induced dissolution or chemical disruption [26] of intrinsic nuclear DNA (chromatin) organization, altering normal metabolic (transcriptional) cascade responses from being activated in response to the hypercaloric microenvironment [14,15]. The ensuing nuclear isolation, chemical disruption and structural dissolution collectively promote the subsequent apoptotic, autolytic demise of cellular organization and structural viability [15], which results in premature cytoatrophy within the affected tissues. The previously noted therapeutic effectiveness of various lipolytic agents [26-28] towards the restoration and maintenance of reproductive tract cytoarchitecture in hypogonadal genotype mutants supports the concept that lipoapoptosis, representing a lipometabolic disruption of cytointegrity within affected db/db cells, effectively compromises reproductive efficiency in experimental models or humans which suffer from Type 2 (NIDDM) diabetes- and obesity-related, hyperlipidemic metabolic (X) syndrome-induced, reproductive dysfunction [24].

In summary, the results of the present studies are the first cyto-chemical and ultrastructural evidence that apoptotic disruption of EE tissue layers in diabetes (db/db) mutant C57BL/KsJ mice occurs coincident with nuclear lipid-infiltration and DNA fragmentation, events that are linked to the hypercaloric metabolic disturbances resulting from progressive cytolipidemia within the female reproductive tract compartments [2,12]. The severity of the cytolipidemia-induced apoptosis was structurally associated with the co-localization of lipid infiltrates into the nuclear compartment of affected cells. Trans-nuclear lipid migration was progressive, migrating from an expanded perinuclear locus, through external nuclear membrane contacts, and ultimate transmembrane diffusion, into the nucleoplasm (Figure 6). The gradual, progressive accumulation of nucleo-lipid depositions eventually disrupted chromatin patterning and distribution, as evidenced by TUNEL-labeled 3'-DNA fragmentation. The gradual lipoapoptotic dissolution of nuclear continuity, and resulting separation from cytoplasmic organelle compartments, promoted pronounced EE cytoatrophy and uterine involution. The hyperlipidemia-induced, apoptotic degradation of intracellular structural integrity and metabolic homeostatic signal cascades [15] compromised reproductive competency, representing common cellular events [5,26], and shared fertility complications [28], experienced by humans and experimental models expressing obesity and Type II (NIDDM) diabetes metabolic syndromes.
